# Evaluation and practice of temporary hemodialysis catheter removal for critically ill patients by Chinese ICU nurses: a multicenter cross-sectional study

**DOI:** 10.3389/fmed.2026.1846635

**Published:** 2026-06-03

**Authors:** Yanting Zhang, Yingying Zeng, Pan Yang, Yuting Huang, Chong Cheng, Jin Li, Xinbo Ding, Jing Ma, Chang Liu, Zhaoyang Li

**Affiliations:** 1 Department of Critical Care Medicine, Hubei Clinical Research Center for Critical Care Medicine, Zhongnan Hospital of Wuhan University, Wuhan, China; 2 Department of Nephrology, Zhongnan Hospital of Wuhan University, Wuhan, Hubei, China

**Keywords:** catheter removal, cross-sectional study, current clinical practice, evaluation factors, intensive care unit, temporary blood purification catheter

## Abstract

**Background:**

Establishing temporary vascular access is essential for critically ill patients undergoing continuous renal replacement therapy (CRRT) in the intensive care unit (ICU). However, such access increases the risk of catheter-related complications, which may exacerbate clinical conditions and even threaten patient survival. This study aimed to investigate current practices and associated evaluation factors regarding temporary hemodialysis catheter removal among Chinese ICU nurses, to clarify why catheters are often retained for prolonged periods after CRRT discontinuation and when removal is typically performed.

**Methods:**

A multicenter, cross-sectional questionnaire survey was conducted among ICU nurses across 30 provinces in China. Data were collected on current practices related to temporary blood purification catheter removal. Chi-square tests were used to compare practices and evaluation factors among nurses with different characteristics. Given the exploratory nature of this study, we did not adjust the significance level for multiple comparisons.

**Results:**

A total of 704 ICU nurses from 32 provincial-level regions in China were included, with 50.14% (353/704) from Central China. The most frequently reported indication for catheter removal was “no longer required for clinical treatment” (394/681, 57.86%). Missing data in ranking questions were handled by pairwise deletion, with percentages calculated only from valid responses. The leading reason for temporary catheter retention after CRRT cessation was “high patient bleeding risk” (284/635, 44.72%). For functionally intact catheters, 65.34% (460/704) of nurses determined removal based on assessment results. The femoral vein was the preferred insertion site (75.74%, 515/680). Daily assessment of catheter retention necessity was supported by 89.35% (629/704) of nurses. Over 80% of respondents judged removal eligibility based on laboratory test results or clinical experience. Physicians were primarily responsible for removal decisions (65.77%, 463/704) and performance of the procedure (66.9%, 471/704). Only 55.4% of nurses had received formal training on catheter removal. Significant differences were observed in removal timing, retention reasons, and management among nurses with different professional characteristics.

**Conclusion:**

ICU nurses in this study demonstrated heterogeneous practices and evaluation criteria for temporary CRRT catheter removal, guided mainly by personal clinical experience and local departmental protocols. Only approximately half of the nurses had received relevant training, and no consensus existed regarding optimal timing and duration for catheter removal. Standardized nursing assessment and recommendation protocols are urgently needed.

## 1 Introduction

Continuous renal replacement therapy (CRRT) is widely utilized as a multi-organ function protection and life-support treatment for critically ill patients (1). Accordingly, establishment of safe and effective temporary vascular access constitutes the foundation for CRRT implementation. Vascular access pathways, often referred to as the “lifeline” for hemodialysis patients, are mainly categorized into temporary and permanent access (2). Owing to the critical condition of ICU patients, most are immobile and require bedside placement to ensure immediate availability and facilitate clinical care; thus, temporary vascular access is routinely selected for CRRT. Temporary blood purification catheters are particularly suitable for emergency hemodialysis, prior to arteriovenous fistula maturation, and as a transitional measure before kidney transplantation, due to their simplicity, rapid placement, reduced pain from repeated punctures, and minimal invasiveness.

However, in clinical practice, we have observed uncertainty regarding the timing and duration of temporary blood purification catheter removal. In some cases, catheters may be retained for an extended period without dialysis until patients are transferred out of the ICU or discharged. Studies have demonstrated that temporary blood purification catheters carry substantial risks of bleeding, infection, and other complications during indwelling, with longer dwell times increasing the likelihood of catheter-related adverse events, which may exacerbate patient conditions and even threaten lives (3, 4). Therefore, early risk assessment and prevention for temporary blood purification catheters are critical. Nevertheless, the perspectives of ICU nurses on temporary blood purification catheter removal remain unclear.

To further develop precise standardized measures, we conducted this multi-regional survey in China to explore current clinical practices and evaluation factors of temporary blood purification catheter removal among Chinese ICU nurses, particularly their views on removal timing, specific time points, and key considerations prior to catheter removal.

## 2 Materials and methods

### 2.1 Study design

This was a multicenter, cross-sectional, large-scale multi-regional survey study.

### 2.2 Participants

This study adopted a convenience sampling method, enrolling ICU nurses from 32 provinces across seven major regions of China (Northeast, East China, North China, Central China, South China, Southwest, and Northwest) between July 2024 and October 2024. Inclusion criteria: ① Holding a valid nursing practice certificate; ② Having at least 6 months of ICU work experience; ③ Voluntary participation in the study; ④ Having experience in CRRT management. Exclusion criteria: ① Absent during the survey period due to sick leave, maternity leave, personal leave, or external training; ② Rotating, advanced training, or intern nurses. The sample size for this cross-sectional survey was set at 5–10 times the number of variables (5). The questionnaire contained 30 items; considering a 20% invalid response rate, the minimum required sample size was determined to be 180–360. All CRRT team members in this study were ICU nursing staff.

### 2.3 Measurements

The questionnaire was developed by our research team based on a literature review (searching Wanfang Database, CNKI, and PubMed from inception to May 2024 using keywords including “blood purification catheter,” “dialysis catheter,” “temporary dialysis catheter,” “central venous catheter,” and “removal” to extract content related to temporary blood purification catheter removal in ICUs) and integrated with clinical experience to draft the initial version.

Fifteen medical and nursing experts with at least 5 years of experience in blood purification and critical care were invited for expert consultation. They evaluated the content validity of the questionnaire using a four-point Likert scale (1 = not relevant to 4 = relevant) and provided revision suggestions. The 15 experts were from nine provinces in China, including two with doctoral degrees, four with master’s degrees, and nine with bachelor’s degrees; five were medical experts and 10 were nursing experts.

Prior to the formal survey, a pilot study was conducted with 20 eligible ICU nurses to solicit feedback on the questionnaire. The average completion time was 5–7 min. Based on their input, question wording was revised appropriately, and explanations were added for certain items and options to enhance clarity and comprehensibility.

To clarify the clinical scenario, all questions regarding catheter removal timing were defined as “after complete discontinuation of CRRT treatment (no further CRRT planned)” rather than after intermittent treatment intervals.

The final questionnaire (see Supplementary Material 1), refined through expert consultation and pilot testing, comprised 30 items divided into two sections. The first section collected respondents’ general information, including region, hospital type, ICU type, gender, age, education level, professional title, nurse type, working years, and CRRT-related work experience. The second section addressed issues related to temporary blood purification catheter removal in critical care settings.

Since this tool was a questionnaire (not a scale) and included ranking and multiple-choice items, Cronbach’s alpha testing was not applicable. Content validity was assessed using the Content Validity Index (CVI), including Item-level CVI (I-CVI) and Scale-level CVI Average (S-CVI/Ave). I-CVI was calculated as the proportion of experts scoring items 3 or 4; I-CVI > 0.78 indicated satisfactory item-level validity. S-CVI/Ave was the mean of all I-CVIs; S-CVI/Ave > 0.9 indicated satisfactory overall scale validity (6, 7). In this study, I-CVI ranged from 0.87 to 1 (>0.78), and S-CVI/Ave was 0.973 (>0.90), confirming good content validity.

Relevant definitions: Clinical assessment: evaluation of bleeding risk, coagulation, vital signs, catheter function, and insertion site. Decision-making: Revised to “assessment and recommendation” (physicians make final decisions). Timing of removal: Defined as after complete discontinuation of CRRT (no further CRRT planned). No national standardized criteria exist and that practices are guided by departmental protocols and personal experience.

### 2.4 Data collection

Data were collected via the Wenjuanxing platform^1^. Research team members who received unified training explained the study purpose and questionnaire completion procedures to participating ICU nurses using standardized instructions. After obtaining informed consent, questionnaires were distributed via WeChat, email, or QR codes. Responses were anonymous. Authors could monitor response progress and export data via the platform backend. Participation was voluntary; nurses provided electronic informed consent by clicking “Confirm” to enter the survey.

### 2.5 Ethics statement

The study adhered strictly to the guidelines outlined in the Declaration of Helsinki (2013, as amended in Fortaleza, Brazil). This study has been reviewed by the Ethics Committee of Zhongnan Hospital of Wuhan University (2023054K). All participants (ICU nurses) provided informed consent.

### 2.6 Quality control

The online questionnaire link was set to non-forwardable to prevent invalid responses. All items were marked as required to avoid missing data. Each IP address was restricted to one response to prevent duplication. Questionnaires with completion time < 3 min or logically inconsistent answers were deemed invalid and excluded. The minimum valid completion time in this study was 3.63 min. Data were entered by one researcher and independently verified by a second researcher. Discrepancies were resolved via discussion or consultation with a third researcher.

### 2.7 Data analysis

Statistical analysis was performed using SPSS 23.0. Categorical data were presented as frequencies and percentages. Comparisons of practices and evaluation factors among nurses with different characteristics were conducted using Chi-square test, Continuity Correction Chi-square test, Likelihood Ratio Chi-square test, or Fisher-Freeman-Halton Exact Test. The significance level was set at α = 0.05, with *P* < 0.05 considered statistically significant. All statistical comparisons were exploratory and intended for hypothesis generation rather than confirmatory inference. Multiple chi-square tests were performed without multiplicity adjustment due to the exploratory design, and the risk of Type I error is acknowledged as a limitation.

For ranking questions, missing data were handled by pairwise deletion—percentages were calculated only from complete valid rankings. We focused on first-rank responses, as they reflect the most prioritized clinical evaluation factor guiding practice; mean rank or weighted scores may dilute the impact of the most critical factor. In this study, “first-rank response” refers to the most important factor selected by respondents.

## 3 Results

### 3.1 Basic information of respondents

A total of 723 questionnaires were distributed, with 704 valid responses collected (response rate: 97.37%). Respondents were ICU nurses from 30 provinces across seven major regions in China, with Central China accounting for the highest proportion (50.14%, 353/704) and Northeast China the lowest (2.84%, 20/704). Most respondents (84.09%, 592/704) worked in tertiary hospitals, and 85.8% (604/704) were from general ICUs. The majority were female (77.56%, 546/704). Approximately half were aged 31–40 years (52.7%, 371/704), and over half (56.53%, 398/704) had ≤5 years of CRRT-related work experience. Detailed characteristics are shown in Table 1.

**TABLE 1:** Basic characteristics of respondents (*N* = 704).

Characteristics	*N* (%)
Regional distribution	
Northeastern China	20 (2.84)
Eastern China	69 (9.80)
Northern China	56 (7.95)
Central China	353 (50.14)
Southern China	53 (7.53)
Southwestern China	58 (8.24)
Northwestern China	95 (13.49)
Hospital types	
Primary level hospitals	14 (1.99)
Secondary level hospitals	98 (13.92)
Tertiary level hospitals	592 (84.09)
ICU types	
General ICU	604 (85.8)
Specialized ICU	100 (14.2)
Age	
20–30 years	278 (39.49)
31–40 years	371 (52.7)
41–50 years	46 (6.53)
>50 years	9 (1.28)
Gender	
Male	158 (22.44)
Female	546 (77.56)
Education level	
Associate degree or below	103 (14.63)
Bachelor’s degree	580 (82.39)
Master’s degree	21 (2.98)
Professional title	
Junior	349 (49.57)
Intermediate	318 (45.17)
Senior	37 (5.26)
Nurse type	
Clinical nurse	478 (67.9)
Blood purification team member	29 (4.12)
Blood purification team leader/responsible leader	34 (4.83)
Specialist nurse	105 (14.91)
Head nurse	58 (8.24)
Years of work experience	
≤5 years	211 (29.97)
6∼10 years	203 (28.84)
11∼20 years	253 (35.94)
≥21 years	37 (5.26)
Years of CRRT-related work experience	
≤5 years	398 (56.53)
6∼10 years	222 (31.53)
11∼20 years	78 (11.08)
≥21 years	6 (0.85)

### 3.2 Current status of temporary blood purification catheter removal in critical care

#### 3.2.1 Timing and specific time points for removal

Respondents were asked to rank indications for temporary blood purification catheter removal. The top indication was “clinical treatment no longer required” (394/681, 57.86%), followed by “catheter malfunction (e.g., malposition, kinking, thrombosis, endothelial hyperplasia)” (394/686, 57.43%), “catheter-related bloodstream infection” (340/681, 49.93%), “uncontrolled bleeding at the insertion site” (330/628, 52.55%), “patient tolerance and compliance with the catheter” (259/555, 46.67%), “conflict with other critical treatments (e.g., ECMO, surgery, long-term catheter replacement)” (215/607, 35.42%), and “economic cost control (nursing and patient costs)” (398/488, 81.56%). Missing data were handled by pairwise deletion, with percentages based on valid responses only.

In terms of the primary reason for catheter retention after complete discontinuation of CRRT treatment, “high patient bleeding risk” ranked first (284/635, 44.72%). When counting the overall endorsement proportion of each influencing factor (not limited to the first priority), “difficult cannulation” accounted for 51.69% (351/679), “departmental management requirements (standard operating procedures)” 50.57% (267/528), “obtaining patient/family consent” 49.44% (267/540), “preparation for sudden clinical deterioration” 39.94% (266/666), and “economic cost control” 64.58% (330/511%). All percentages were calculated based on valid responses after pairwise deletion of missing ranking data.

For functionally intact catheters, 65.34% (460/704) of nurses decided removal based on assessment results. Additionally, 12.64% removed catheters only upon ICU transfer, discharge, or death; 9.66% removed catheters within 72 h, and 6.39% within 24 h after complete CRRT cessation.

#### 3.2.2 Assessment before catheter removal

In total, 75.74% (515/680) of ICU clinicians selected the femoral vein as the preferred insertion site for temporary blood purification catheters. The second most common site was the right internal jugular vein (332/562, 59.07%), followed by the left internal jugular vein (221/473, 46.72%) and subclavian vein (235/382, 61.52%).

A total of 89.35% (629/704) of nurses supported daily assessment of catheter retention necessity for functional catheters. Approximately half (46.88%, 330/704) recommended care every 12–24 h for retained catheters after treatment cessation to preserve function, while 27.98% (197/704) preferred situation-based care (e.g., for circuit blood reflux or puncture site bleeding). Over 90% of respondents agreed that vital signs, bleeding/coagulation risk, insertion site condition, and catheter integrity should be evaluated before removal to ensure safety. More than 80% judged removal eligibility using laboratory results or clinical experience. Details are shown in Table 2.

**TABLE 2:** Intensive care unit (ICU) nurses’ current status of pre-removal assessment for temporary blood purification catheters (*N* = 704).

Item	*N* (%)
Frequency of assessing retention necessity for functional catheters	
Daily assessment	629 (89.35)
Every 48 h	40 (5.68)
Every 72 h	29 (4.12)
Weekly	6 (0.85)
Care frequency for retained catheters after treatment cessation	
12–24 h	330 (46.88)
48 h	108 (15.34)
96 h	20 (2.84)
7 days	24 (3.41)
Condition-based care (e.g., dialysis circuit backflow or bleeding at the puncture site)	197 (27.98)
At next blood purification treatment	25 (3.55)
Assessment content	
Vital signs	651 (92.47)
Bleeding/coagulation risk (coagulation tests, platelet count, no high-dose anticoagulation)	690 (98.01)
Achievement of treatment goals (urine output, internal environment, etc.)	684 (97.16)
Insertion site condition (skin integrity, bleeding, infection signs)	678 (96.31)
Insertion depth and external markings of the catheter	620 (88.07)
Patency of the catheter (e.g., presence of thrombus)	655 (93.04)
Assessment methods	
Ultrasound assessment	459 (65.2)
Laboratory test results	571 (81.11)
Experiential assessment (inspection, palpation, aspiration check)	571 (81.11)
Patient symptom inquiry	256 (36.36)

#### 3.2.3 Intra- and post-removal management

For catheter removal, patients were placed in the supine position for internal jugular vein catheters (45.03%, 317/704) and femoral vein catheters (74.57%, 525/704). Removal decisions were primarily made by physicians (65.77%, 463/704), followed by multidisciplinary medical-nursing collaboration (28.13%, 198/704); only 0.85% (6/704) were nurse-led decisions. The procedure was mostly performed by physicians (66.9%, 471/704).

After removal, 53.41% (376/704) used manual or device-assisted pressure for hemostasis, and 81.68% (575/704) covered the site with sterile gauze or surgical dressings. Most nurses (79.26%, 558/704) reported no intra-procedure complications. The most common post-removal complications were hematoma (74.72%, 526/704), persistent bleeding (65.77%, 463/704), and medical adhesive-related skin injury (61.51%, 433/704). Details are presented in Table 3.

**TABLE 3:** Intensive care unit (ICU) nurses’ handling during and after removal of temporary blood purification catheters (*N* = 704).

Item	*N* (%)
Patient position for internal jugular vein catheter removal	
Trendelenburg position (head low and supine)	109 (15.48)
Supine position	317 (45.03)
Semi-recumbent position	200 (28.41)
No specific position	76 (10.8)
Other positions	2 (0.28)
Patient position for femoral vein catheter removal	
Trendelenburg position (head low and supine)	34 (4.83)
Supine position	525 (74.57)
Semi-recumbent position	94 (13.35)
Not specified / routine position without special consideration	51 (7.24)
Decision-maker for safe catheter removal	
Doctor	463 (65.77)
Nurse	6 (0.85)
Vascular access team/CRRT team member	37 (5.26)
Medical-nursing collaborative assessment	198 (28.13)
Intra-procedure complications	
Catheter-related: difficulty in catheter removal/catheter breakage	187 (26.56)
Condition-related: transient hypotension/vasovagal response/pulmonary embolism/difficulty breathing/cardiac arrest/pneumothorax/air embolism	172 (24.43)
No complications	558 (79.26)
Healthcare provider performing catheter removal	
Doctor	471 (66.9)
Nurse	99 (14.06)
Vascular access team/CRRT team member	41 (5.82)
Medical-nursing collaboration	93 (13.21)
Post-removal hemostasis method	
Manual/device pressure	376 (53.41)
Suture	4 (0.57)
Compressive bandaging	161 (22.87)
Decision on pressure application method based on assessment results	163 (23.15)
Post-removal puncture site care	
Sterile gauze/surgical dressing	575 (81.68)
Sterile/transparent dressing	120 (17.05)
No coverage after pressure	9 (1.28)
Post-removal pressure duration	
5–10 min	66 (9.38)
10–20 min	203 (28.84)
20–30 min	134 (19.03)
>30 min	111 (15.77)
Decide the pressure application time based on assessment results	190 (26.99)
Patient immobilization time after catheter removal	
≤30 min	49 (6.96)
30 min∼1 h	176 (25)
1–2 h	126 (17.9)
>2 h	162 (23.01)
No specific requirements for immobilization time	191 (27.13)
Post-removal complications	
Withdrawal distress syndrome	196 (27.84)
Hematoma	526 (74.72)
Persistent bleeding (>10 min)	463 (65.77)
Thrombosis	225 (31.96)
Puncture site infection	286 (40.63)
Adhesive tape tearing injuries/medical adhesive-related skin injuries at the puncture site	433 (61.51)

Pain, vasovagal response, air embolism, thrombus dislodgement, and infection, with the status noted as: “A list based on a literature review and expert consultation; not exhaustive.” Withdrawal distress syndrome: the clinical process of complications such as hypertension and tachycardia occurring after catheter removal is called central venous catheter withdrawal distress syndrome.

#### 3.2.4 ICU management of temporary blood purification catheters

Only 55.4% (390/704) of nurses had received formal training on catheter removal, while 70.6% (497/704) worked in ICUs with standardized catheter removal protocols. Responses reflected actual daily practice rather than required protocols; subgroup analysis by unit was not performed due to the large number of participating centers and focus on individual nurse perspectives.

### 3.3 Comparison of views on the removal of temporary blood purification catheters among ICU nurses with different characteristics

Exploratory analysis revealed significant differences in the ranking of catheter removal timing among nurses from different regions, ages, genders, education levels, professional titles, working years, and CRRT-related experience (see Supplementary Tables 1, 4–7, 9, 10). Significant differences were also found in the ranking of reasons for catheter retention across regions, hospital types, ages, education levels, professional titles, working years, and CRRT-related experience (Supplemental Material 2, Supplementary Tables 1, 2, 4, 6, 7, 9, 10). For removal timing of functional catheters, significant differences were observed only by education level, professional title, and working years (Supplementary Tables 6, 7, 9). Detailed comparative results are provided in Supplementary Tables 1–10.

## 4 Discussion

Continuous renal replacement therapy is a common and vital treatment for critically ill ICU patients. Catheter-related infection is a frequent complication after CRRT catheter placement, with the risk increasing with longer dwell time. Prolonged indwelling can lead to catheter-related bloodstream infections and colonization, elevating patient mortality and hospitalization costs (8, 9). Extended catheter retention is also associated with higher rates of bleeding, hematoma, and thrombosis, compromising patient safety and clinical outcomes (3, 4). This survey aimed to clarify why ICU nurses retain catheters long after CRRT cessation and the typical timing of removal.

Our results showed that the top indication for catheter removal was “no longer required for clinical treatment.” However, catheter malfunction, catheter-related bloodstream infection, and uncontrolled insertion site bleeding ranked second to fourth. The Chinese *Expert Consensus on Infection Prevention and Control for Continuous Renal Replacement Therapy in Hospitals* (10) recommends catheter removal as soon as it is no longer needed (no specific time frame), with a maximum dwell time of 14 days with proper sterile technique. Previous studies reported mean catheter dwell times of (21.4 ± 4.61) days (11) and a median of 14 (7–30) days (12) in Chinese ICUs. Chen et al. (13) identified catheter dwell time > 7 days as a risk factor for catheter-related bloodstream infection in acute kidney injury patients receiving CRRT. Thus, CRRT catheter dwell times in China are generally prolonged, exceeding consensus recommendations and increasing complication risks and worsening patient outcomes (8, 9, 13).

In clinical practice, most nurses believed catheter malfunction could often be reversed by repositioning or thrombolysis, and catheter-related bloodstream infection may be -managed with antimicrobial therapy before removal decisions. These approaches may reduce treatment costs and alleviate patient economic burden. Notably, only 9.66% and 6.39% of nurses removed functional catheters within 72 and 24 h of CRRT cessation, respectively; most relied on assessment results.

The UK *Recommendation for the Safe Removal of a Temporary Femoral Dialysis Line* (14) advises coagulation and platelet testing before removal and avoidance of high-dose anticoagulation, consistent with our finding that “high bleeding risk” was the top reason for catheter retention. Over 90% of nurses assessed vital signs, bleeding/coagulation risk, insertion site condition, and catheter integrity before removal, and over 80% used laboratory results or clinical experience to guide decisions, all aimed at preventing bleeding-related adverse events.

Catheter insertion site selection may affect RRT efficiency and patient safety (15). Common sites include the internal jugular, subclavian, and femoral veins, with no universal consensus. Western centers prefer internal jugular or subclavian veins (15–17), while 75.74% of Chinese clinicians in our study chose the femoral vein. A meta-analysis by Arvaniti et al. (18) found that internal jugular and subclavian veins carried lower catheter-related infection risks than the femoral vein, with the subclavian vein being optimal when colonization risk is considered. Thus, controversy remains regarding optimal insertion site selection.

Expert consensus (10) recommends sterile gauze or transparent dressings for insertion site care, with gauze for heavy drainage and dressing changes every 5–7 days for transparent dressings and at least every 2 days for gauze, consistent with our findings. No consensus exists regarding patient position during removal; our results confirm supine positioning as standard practice in China.

Comparisons by nurse characteristics revealed significant differences in removal timing, retention reasons, and functional catheter removal timing by professional title, education level, working years, and CRRT-related experience. In China, intermediate and senior titles generally reflect extensive clinical experience. Junior-titled nurses prioritized patient experience (tolerance/compliance) and economic considerations, likely due to stronger empathy. Senior nurses focused more on treatment outcomes, which may reflect reduced empathy over time (19–22). The small sample of senior nurses (*n* = 39) may have introduced bias. Thus, results should be interpreted cautiously, and future studies should expand sample sizes.

## 5 Limitations

To our knowledge, this is the first study investigating ICU nurses’ practices and evaluation factors for temporary blood purification catheter removal. It clarifies nurses’ perspectives on removal timing, duration, and key pre-removal considerations, providing a foundation for standardized protocols and guidelines.

However, this study has several limitations. Firstly, convenience sampling was used, with uneven regional distribution (Central China: 50.14%), which may reduce generalizability and affect regional subgroup reliability. Secondly, the online questionnaire relied on multiple-choice items and did not explore in-depth clinical challenges or solutions. Thirdly, numerous chi-square tests were performed without multiplicity adjustment (e.g., Bonferroni correction), which substantially increases the risk of Type I error. All significant findings are exploratory and hypothesis-generating rather than confirmatory conclusions. Therefore, the results should be interpreted with caution and require validation in future prospective studies. Fourthly, the study lacked objective clinical outcome data (e.g., actual dwell time, complication rates, patient prognosis) and relied on subjective nurse self-report, which may be affected by recall or response bias. The distribution method (via hospital WeChat groups) may have introduced social desirability bias or perceived administrative pressure, despite anonymity being assured. Fifth, the study relies entirely on self-reported data from ICU nurses, without direct observation, audit, or medical record verification. This approach carries a risk of recall bias, as respondents may not accurately remember detailed clinical practices. In addition, social desirability bias may exist, whereby participants may report ideal or guideline-consistent practices rather than their actual daily behavior. Although the questionnaire explicitly asked for responses based on actual daily practice rather than required protocols, potential bias cannot be eliminated. Therefore, all findings reflect reported practice rather than objectively confirmed clinical reality. Future qualitative research may explore nurses’ in-depth perspectives, and prospective studies with objective clinical data are needed to validate findings. Multivariable regression or structural equation modeling could be used to identify predictive factors.

In addition, the participants were predominantly from tertiary hospitals, while the sample size of primary and secondary hospitals was small. Therefore, the findings mainly reflect the current situation of ICU nurses in tertiary hospitals in China, and cannot fully represent the actual practice status of primary and secondary hospitals. Over-generalization of the research results to all levels of hospitals should be avoided. Meanwhile, the regional distribution of respondents was uneven, with Central China accounting for the highest proportion, which may affect the representativeness of regional subgroup analysis results.

## 6 Conclusion

This multi-regional cross-sectional study describes current nursing assessment and clinical practices regarding temporary hemodialysis catheter removal among Chinese ICU nurses. Wide variation exists in catheter removal timing, assessment criteria, and clinical management, which are largely guided by individual clinical experience and local departmental protocols rather than standardized national guidelines. Only approximately half of ICU nurses have received formal training related to catheter removal assessment and management.

Future prospective studies with objective clinical outcomes and validated measurement tools are needed to confirm these findings, explore associations between practices and complication rates, and develop evidence-based standards for temporary CRRT catheter removal in critically ill patients.

## Data Availability

The raw data supporting the conclusions of this article will be made available by the authors, without undue reservation.

## Footnotes

1
https://www.wjx.cn/vm/tep8ULH.aspx#
