# Supplementary material for: Evaluation and practice of temporary hemodialysis catheter removal for critically ill patients by Chinese ICU nurses: a multicenter cross-sectional study
Source: Front Med (Lausanne). 2026 Jun 3;13:1846635. doi: 10.3389/fmed.2026.1846635 (PMC13272147; doi:10.3389/fmed.2026.1846635)
Supplement: Supplementary file 1 [file Table_1.docx]

**Survey on the Current Status of Critical Care Nurses' Removal of Temporary Blood Purification Catheters in Chinese Intensive Care Units**

**Part One: Basic Characteristics**

1. Your hospital's location:

- Northeast region

- East China region

- North China region

- Central China region

- South China region

- Southwest region

- Northwest region

2. Type of your hospital:

- First-level hospital

- Second-level hospital

- Third-level hospital

3. Your department:

- General ICU (Intensive Care Unit)

- Specialized ICU

4. Your age:

- 20-25 years old

- 26-30 years old

- 31-39 years old

- 40 years old and above

5. Your years of work experience:

- 5 years or less

- 6-10 years

- 11-20 years

- 21 years or more

6. Years of experience in CRRT (Continuous Renal Replacement Therapy) related work:

- 5 years or less

- 6-10 years

- 11-20 years

- 21 years or more

7. Your gender:

- Male

- Female

8. Your highest level of education:

- Associate degree or lower

- Bachelor's degree

- Master's degree or higher

9. Your professional title:

- Junior title

- Intermediate title

- Senior title

10. Your position:

- Clinical nurse

- Member of the blood purification team

- Leader/responsible person of the blood purification team

- Specialist nurse

- Head nurse

**Part Two: Current Status of Temporary Blood Purification Catheter Removal**

| **01** | **In which of the following situations would you choose to remove a dialysis catheter? (Rank according to primary factors)**  - Clinical treatment is no longer necessary  - Catheter malfunction (such as abnormal catheter position, catheter twisting, catheter thrombosis, catheter endothelial hyperplasia, etc.)  - Concurrent catheter-related bloodstream infection  - Hemorrhage around the catheter insertion site with failed hemostasis  - Patient's tolerance and compliance with the catheter  - Conflict with other crucial treatment methods (such as ECMO therapy, surgical procedures, replacement of long-term dialysis catheters, etc.)  - Economic cost control (such as nursing costs, patient costs) |
| --- | --- |
| **02** | **After the end of CRRT treatment, in which situation would you choose to temporarily retain the catheter instead of removing it? (Rank according to primary factors)**  - The patient has a high risk of bleeding  - Difficulty in catheter placement  - To be ready for sudden changes in the patient's condition  - After obtaining consent from the patient/family  - Department management requirements (established operational standards)  - Economic cost control (such as nursing costs, patient costs) |
| **03** | **For temporary dialysis catheters, when the catheter function is intact, at what time would your department choose to remove it?**  - Immediately after discontinuing use  - Within 24 hours of temporary use  - Within 72 hours of discontinuing use  - After 7 days of discontinuing use  - Upon transfer out of ICU/discharge from hospital/death |
| **04** | **In the case where the catheter function is intact, how often does your department assess the necessity for catheter retention?**  - Daily assessment  - Every 48 hours  - Every 72 hours  - Weekly/per occasion |
| **05** | **Before catheter removal, what content would you evaluate? (Multiple choices)**  - Vital signs  - Risk of bleeding or clotting (such as coagulation function and platelet test indicators, confirming no high-level anticoagulant treatment)  - Whether the therapeutic goals have been achieved (urine output, internal environment)  - Condition of the catheter insertion site (skin integrity, presence of bleeding/oozing, signs of infection, etc.)  - Depth of catheter insertion and external leakage scale  - Catheter patency status (e.g., presence of thrombosis) |
| **06** | **In your department, who determines the decision for safe catheter removal?**  - Doctor  - Nurse  - Vascular access team/CRRT team members  - Medical and nursing staff collaborate in decision-making |
| **07** | **In your department, who completes the catheter removal procedure?**  - Doctor  - Nurse  - Vascular access team/CRRT team members  - Medical and nursing staff collaborate in the procedure |
| **08** | **In your department, how do you assess whether to remove a dialysis catheter?**  - Ultrasound assessment  - Laboratory tests  - Empirical evaluation (visual inspection, palpation, retraction check, etc.)  - Asking the patient's chief complaint |
| **09** | **In your department, what are the common sites for temporary dialysis catheter placement?**  - Left internal jugular vein  - Right internal jugular vein  - Femoral vein  - Subclavian vein |
| **10** | **During the removal of an internal jugular vein dialysis catheter, what position do you adopt?**  - Low head supine position  - Supine position  - Semi-recumbent position  - Position not considered  - Other positions (please specify)________ |
| **11** | **During the removal of a femoral vein dialysis catheter, what position do you adopt?**  - Low head supine position  - Supine position  - Semi-recumbent position  - Position not considered  - Other positions (please specify)________ |
| **12** | **After catheter removal, how is the puncture site managed?**  - Compression (manual/pressor)  - Suturing  - Pressure dressing  - Decision on compression method based on assessment results |
| **13** | **After catheter removal, how long do you typically apply pressure to the puncture site?**  - 5-10 minutes  - 10-20 minutes  - 20-30 minutes  - >30 minutes  - Decision on duration of pressure application based on assessment results |
| **14** | **In your department, how long is the patient typically required to remain immobilized after catheter removal?**  - ≤30 minutes  - 30 minutes to 1 hour  - 1-2 hours  - >2 hours |
| **15** | **After catheter removal, how is the puncture site managed?**  - Covered with sterile gauze/surgical dressing  - Sealed with sterile/transparent dressing  - No covering after compression |
| **16** | **During catheter removal, what complications have you encountered in patients?**  - Catheter-related: difficulty in catheter removal/catheter breakage  - Condition-related: transient shock/pulmonary embolism/difficulty breathing/cardiac arrest/pneumothorax/air embolism  - No complications occurred |
| **17** | **After catheter removal, what complications have you encountered in patients?**  - Catheter removal distress syndrome (the clinical process of complications such as hypertension and tachycardia occurring after central venous catheter removal is known as catheter removal distress syndrome)  - Hematoma  - Persistent bleeding (bleeding time >10 minutes)  - Thrombosis  - Infection at the puncture site  - Skin injuries from adhesive tape tearing/medical adhesive-related skin injury |
| **18** | **Have you received training on catheter removal procedures related to dialysis?**  - Yes  - No |
| **19** | **Does your department have a standardized, regulated catheter removal process?**  - Yes  - No |
| **20** | **How often is the maintenance carried out for a dialysis catheter that remains temporarily in place after dialysis treatment?**  - 12-24 hours  - 48 hours  - 96 hours  - 7 days  - As needed (e.g., dialysis circuit backflow or bleeding at the puncture site)  - During the next blood purification treatment |
